# Can we reliably use pulse pressure as a surrogate for stroke volume? Physiological background and potential clinical implications for shock resuscitation

**DOI:** 10.1186/s13054-025-05490-9

**Published:** 2025-06-19

**Authors:** Zbigniew Putowski, Jean-Louis Teboul, Ricardo Castro, Denis Chemla, Jonatan Oras, Sebastian Morales, Eduardo Kattan, Glenn Hernandez

**Affiliations:** 1 Centre for Intensive and Perioperative Care, Jagiellonian University Medical College, Cracow, Poland; 2 Faculté de Médecine Paris-Saclay, Université Paris-Saclay, Le Kremlin-Bicêtre, Paris, France; 3 Departamento de Medicina Intensiva, Facultad de Medicina, Pontificia Universidad Católica de Chile, Santiago, Chile; 4 INSERM UMRS 999, Hôpital Marie Lannelongue, Le Plessis-Robinson, Paris, France; 5 Department of Anaesthesiology and Intensive Care Medicine, Clinical Sciences, University of Gothenburg and Sahlgrenska University Hospital, Gothenburg, Sweden; 6 Departamento de Medicina Intensiva, Facultad de Medicina, Pontificia Universidad Católica de Chile, Avenida Diagonal Paraguay 362, Santiago, 6510260 Chile

**Keywords:** Stroke volume, Pulse pressure, Hemodynamic monitoring, Shock resuscitation

## Abstract

In critically ill patients, early hemodynamic assessment is essential for guiding shock resuscitation. While cardiac output (CO) is a key indicator of circulatory status, its measurement is often limited by technical and practical constraints. This perspective explores the physiological and clinical relevance of pulse pressure (PP) as a potential surrogate for stroke volume (SV), emphasizing its accessibility at the bedside. The paper discusses how factors such as arterial compliance, vascular tone, and pulse wave amplification influence the PP-SV relationship, often complicating interpretation in acute and complex hemodynamic states. It also examines the effects of vasopressors, vascular decoupling, and catheter site on PP measurements, particularly in septic shock. Despite its limitations, the review highlights how peripheral PP, when carefully interpreted, may aid in identifying low SV and guiding early resuscitation strategies.

## Background

Critically ill patients often exhibit cardiovascular disturbances that impair oxygen delivery, leading to organ dysfunction. Monitoring tissue perfusion and identifying predominant hemodynamic patterns are essential clinical objectives for guiding tailored resuscitative strategies [1]. This approach is particularly important in the early stages of disease, when timely interventions can significantly impact outcomes [2]. Cardiac output (CO) monitoring is central to hemodynamic assessment, offering insights into global blood flow and distinct hemodynamic profiles [3]. However, its widespread use is limited by high costs, invasiveness, and the need for specialized training, particularly in low- and middle-income countries [4].

Invasive arterial pressure monitoring, a standard technique in critically ill patients, provides diverse relevant signals beyond mean arterial pressure (MAP) [5] including systolic arterial pressure (SAP), diastolic arterial pressure (DAP), and pulse pressure (PP) among others. A key question is if, and to what extent any of these variables may aid clinicians in predicting stroke volume (SV) to decide on initial resuscitative interventions before more advanced hemodynamic monitoring, including echocardiography, has been placed or performed. MAP is considered as the global perfusion pressure and exerts the greatest influence on blood flow autoregulation within organs [6]. However, nor an isolated MAP value, physiologically determined by both SV and systemic vascular resistance, or its changes after an acute hemodynamic intervention may predict SV. Indeed, MAP changes are conditioned by the status of dynamic elastance after a fluid challenge, while some patients increase SV without proportional changes in MAP or vice versa [7].

On the other hand, SAP relates to the work that the left ventricle must perform to generate an adequate SV and reflects the interplay between cardiac performance and heart rate, the buffer mechanical function of the aorta, and peripheral resistances. Therefore, SAP cannot be directly linked with SV. Conversely, DAP is mainly determined by vascular tone and remains nearly constant from the ascending aorta to the peripheral vessels [5]. Evaluation of the loss of vascular tone or vasoplegia through the severity of diastolic hypotension could have profound implications on therapeutic decisions, such as early use of norepinephrine in septic shock [8].

Thus, it appears that the signal that may offer more insights into the status of SV is pulse pressure, as discussed below. However, the PP-SV relationship is influenced by complex physiological factors, complicating its bedside interpretation. This review explores the physiological basis of the PP-SV relationship, the factors affecting it, and providing perspectives on the clinical utility in hemodynamic profiling and shock resuscitation.

## Physiological principles of pulse pressure

PP, defined as the difference between SAP and DAP, reflects the heart’s energy output to the arterial system [9]. Intuitively, PP increases when SV increases, as larger volumes must be accommodated by a greater pressure rise in the arteries. Theoretically, SV should be then directly proportional to PP [10]. Indeed, this can be easily exemplified by the PP responses observed during the Valsalva maneuver. During the strain phase, the decrease in venous return leads to a reduction in SV, resulting in a decline in PP. Conversely, during the release phase, a marked increase in venous return leads to a substantial rise in SV, being reflected in higher PP. Notwithstanding, SV is not the only determinant of PP. The relationship is also modulated by arterial characteristics—especially their mechanical properties and how they influence wave propagation [11]. These factors (Table 1) are critical in understanding how pressure is generated and sustained in the circulatory system.

**Table 1:** Main components affecting arterial contour and pulse pressure [11]

Components	Factors
Incident pressure wave	♣ Compliance of aorta ♣ Stroke volume ♣ Velocity of ejection ♣ Cardiac efficiency (preload, afterload, heart rate, contractility, pattern of ejection, impedance to ejection)
Reflected pressure wave	♣ Arterial stiffness ♣ Mean arterial pressure ♣ Degree of vasoconstriction and vasomotor tone ♣ Distance from reflecting sites ♣ Timing of arrival in cardiac cycle ♣ Duration of ejection ♣ Sex ♣ Body height

Aortic and arterial compliance play a crucial role in the process of the volume-to-pressure transformation. Compliance (C) represents the ability of central arteries to buffer the sudden influx of blood, preventing excessive rises in left ventricular ejection pressure [12]. When ‘C’ decreases—typically due to arterial stiffening—the arteries’ buffering capacity diminishes. This results in a higher PP, as stiffer arteries require the ventricle to produce stronger contraction for the same SV. This occurs in many old individuals and is commonly known as isolated systolic hypertension, which is thus characterized by increased aortic SAP with non-increased DAP [13]. On the other hand, a low aortic PP would indicate a lower-than-normal SV, as abnormally low arterial stiffness is rare [14]. Based on the above considerations, a normal aortic PP would suggest either a normal SV with normal arterial stiffness or a reduced SV with increased arterial stiffness. Interpreting aortic blood pressure values would thus be helpful to identify some mechanisms underlying acute circulatory failure. For instance, low MAP would indicate low perfusion pressure; low DAP [15] would reflect low arterial tone (especially when adjusted for heart rate) [16]; and low aortic PP would suggest low SV, particularly in patients with stiff arteries including older ones. Unfortunately, measurements of aortic blood pressure are rarely available.

In clinical practice, PP is measured peripherally, usually at brachial, radial or femoral arteries. Importantly, peripheral PP values tend to significantly differ from those measured proximally. It is due to the so-called pulse wave amplification (PWA) [17]. PWA is traditionally explained by the reflection of arterial pressure waves at sites of impedance mismatch, such as vascular bifurcations. The cumulative effect of these reflected waves creates a retrograde pressure wave traveling back to the aorta. The backward pressure wave combines with the forward pressure wave related to the blood ejection, together producing the final aortic pressure waveform. In young and healthy individuals, this backward wave peaks at the beginning of diastole and elevates diastolic aortic pressure. When arteries are stiffer or reflection sites are closer (e.g., due to vasoconstriction), the backward pressure wave returns more quickly to the aorta, causing it to overlap with the forward pressure wave primarily during systole. This results in a final aortic pressure wave with a systolic peak that exceeds the maximum pressure of the forward aortic pressure wave.

**Fig. 1:**
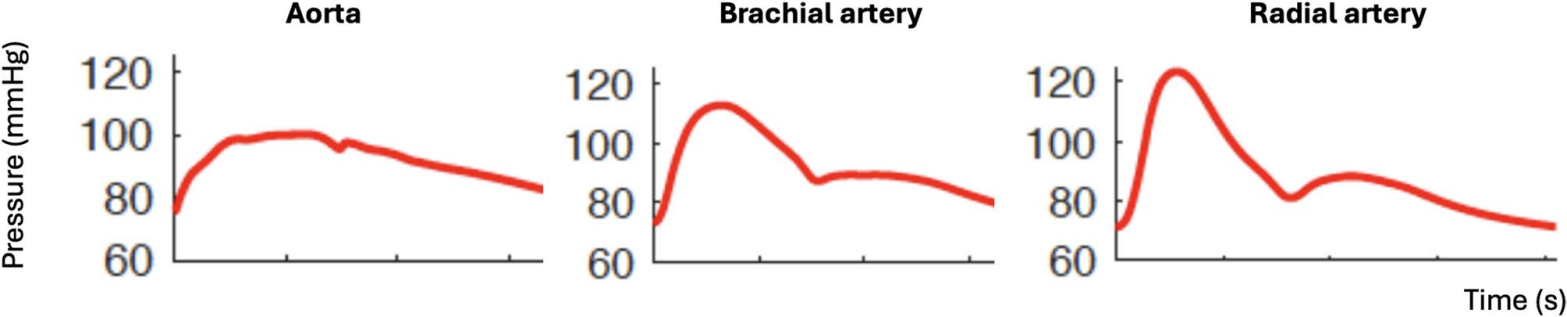
A schematic representation of pulse wave amplification phenomenon

To well understand PWA, it is crucial to note that the forward pressure wave, generated by the interaction between left ventricular ejection and aortic wall compliance, propagates through an arterial tree that becomes progressively stiffer and narrower. This causes the morphology of the arterial pressure waveform to change as it moves from the aorta to the periphery (Fig. 1). Consequently, peripheral SAP is higher than the peak of the forward aortic pressure wave, while peripheral DAP is only slightly lower than the nadir of the aortic pressure wave. Therefore, PWA results in higher SAP (around 10–15 mmHg on average), slightly lower DAP (around 1 mmHg on average), and higher PP (around 10–15 mmHg on average) in peripheral arteries compared to the aorta. Because the femoral artery is near the aorta, PP in the femoral artery closely matches aortic PP and is lower than brachial PP, which in turn is lower than radial PP. Importantly, the difference between aortic and peripheral PP can be influenced by the timing of the backward pressure wave’s arrival at the aorta. When the backward pressure wave returns fast, peaking during systole, it augments aortic SAP above the peak of the forward pressure wave, reducing the difference between peripheral SAP (influenced by the amplification of the forward pressure wave) and aortic SAP. Of note, increased aortic SAP due to PWA inflicts greater strain on the contracting left ventricle [18]. The strengthening of PWA occurs mainly in cases of vasoconstriction, higher arterial resistance and shorter arterial trees (e.g., in smaller individuals).

Recent data from longitudinal studies showed that the development of wide aortic PP with aging (after midlife) is strongly associated with proximal aortic stiffening resulting in larger forward pressure wave amplitude, whereas PWA has a lesser contribution [19]. Accordingly, in individuals older than 60 years, even healthy but with stiff arteries due to aging, the difference between peripheral PP and aortic PP is reduced compared to young individuals (around 0-5mmHg vs. around 10–15 mmHg on average) [20]. Therefore, in old patients and even more in those suffering from chronic hypertension or diabetes, what is measured in periphery could eventually reflect what it exists in the aorta. Nevertheless, when marked, PWA affects the value of PP, making it less correlated with SV, and more dependent on the overall condition of the cardiovascular system and technical aspects of measurement.

## Pathophysiological and technical aspects of PP-SV relationship

**Fig. 2:**
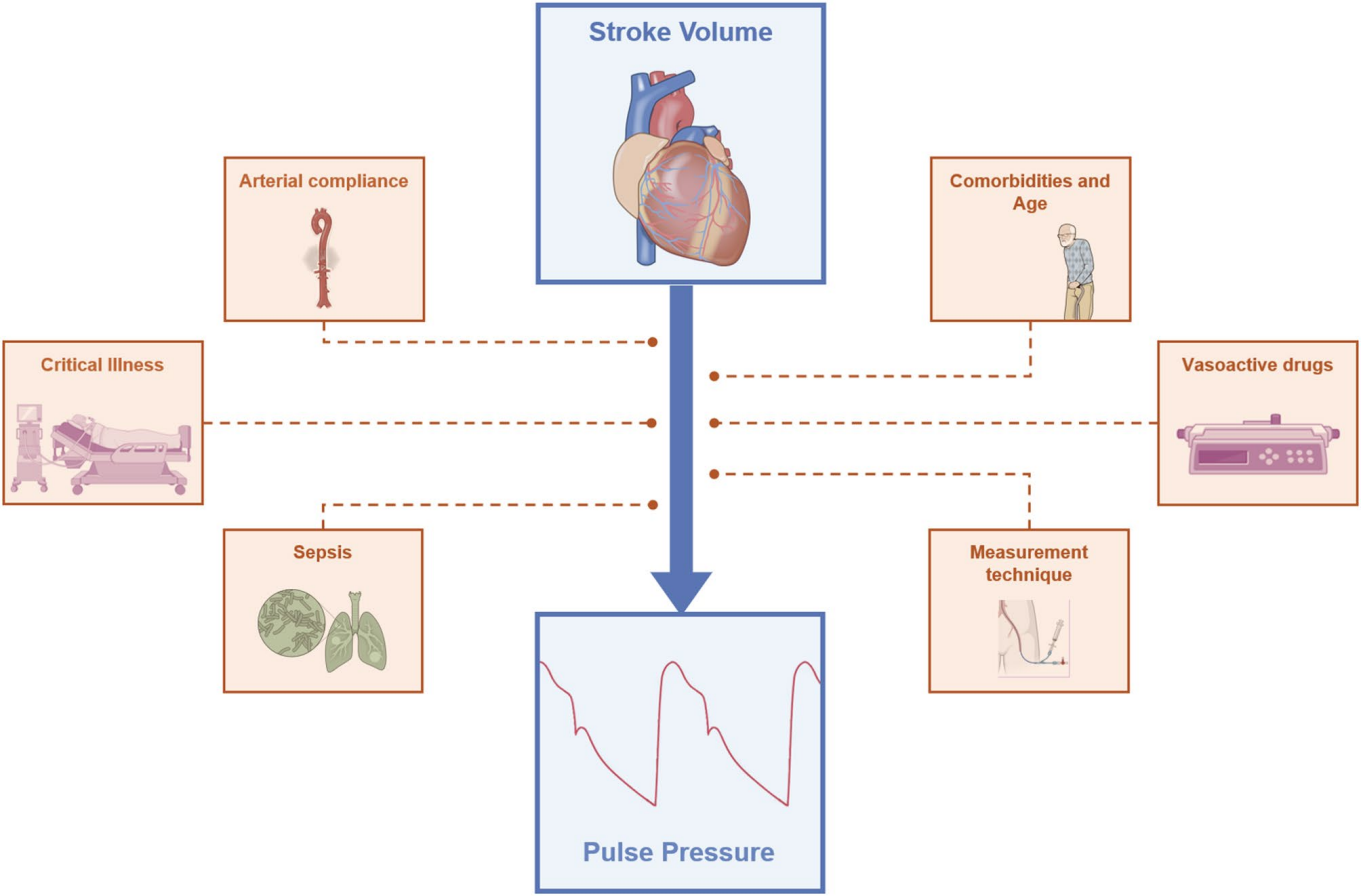
Schematic representation of the main variables that impact the relationship between stroke volume and pulse pressure

As outlined above, the relationship between aortic PP and SV could be direct, particularly beat-to-beat conditions, where C is expected to remain constant over the MAP range under study. Consequently, aortic PP variations are anticipated to primarily reflect SV variations. Naturally, this relationship is modulated by various additional factors, including arterial compliance, PWA, vasomotor tone, and the anatomical site of arterial pressure measurement (Fig. 2). If these factors become profoundly altered, the relationship between changes in SV and corresponding variation in aortic and – especially – peripheral PP become less predictable, as is likely the case in severe circulatory dysfunction [21, 22]. This was evidenced by one study in which SV and arterial stiffness explained 90% of the variability in peripheral PP in older patients [23]. Among these patients, SV alone accounted for 49% of PP variability when arterial stiffness was considered [20, 24].

### Sepsis

**Fig. 3:**
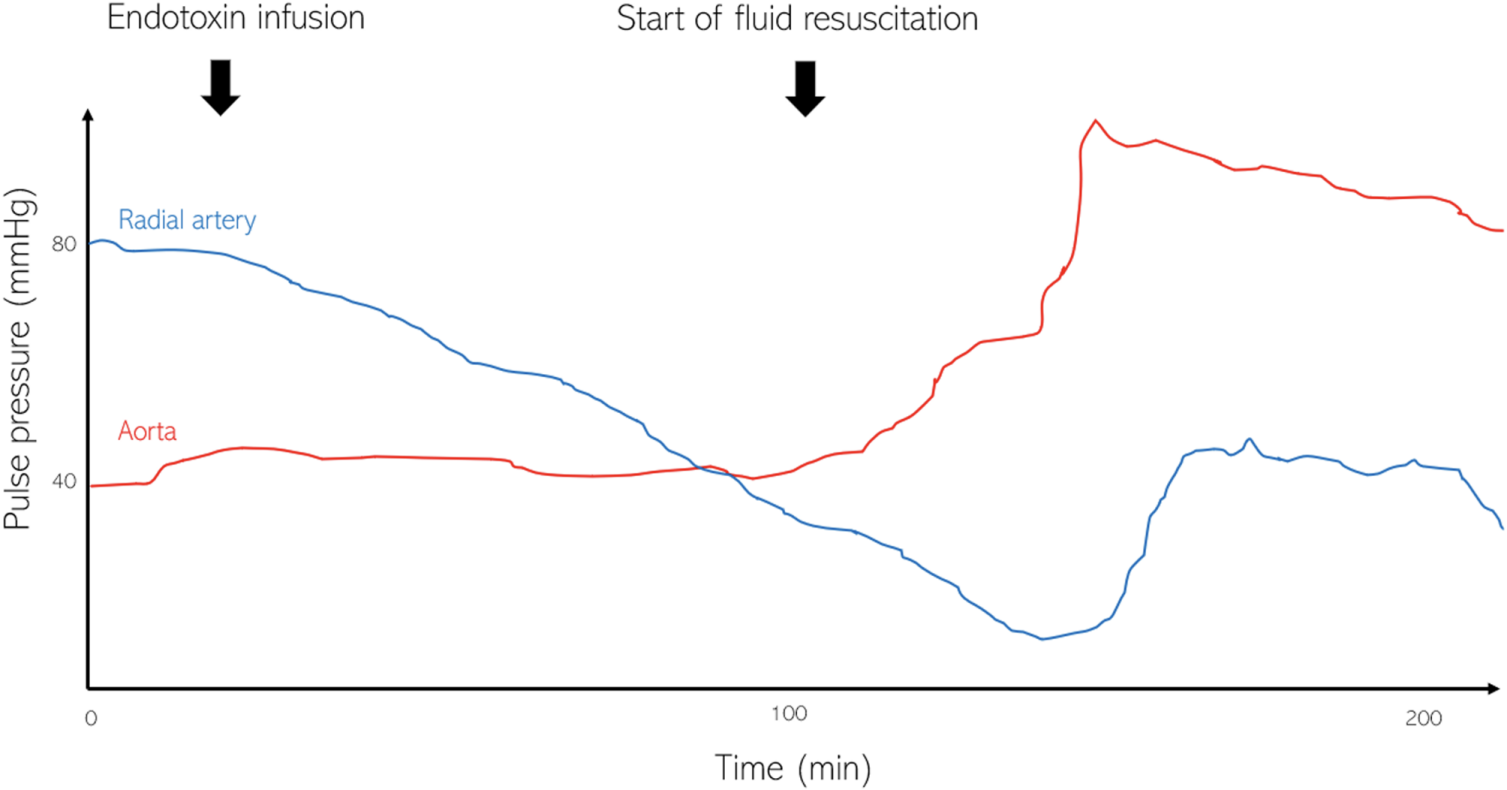
Vascular decoupling in septic shock [25]

Experimental studies using porcine models showed that endotoxin-induced vasodilation improves arterial compliance, particularly within distal arteries [25]. This phenomenon leads to what is termed “vascular decoupling”, where vasodilation reduces the intensity of reflected waves, resulting in diminished or even reversed PWA. Consequently, in such a scenario, the highest PP can be observed in the aorta and the lowest PP in the periphery (Fig. 3). Another porcine experimental study corroborated these observations, demonstrating that shock induces a loss of PWA [26]. This may explain why fluid expansion has been observed to significantly increase SV without a corresponding direct increase in PP [7]. The above models suggest that in septic shock, even a high SV may not result in a proportionally high PP.

### Vasoactive drugs

Vasoactive agents also influence the relationship between PP and SV by modifying arterial tone and resistance. Since vasoactive agents are the cornerstone of septic shock resuscitation, their effects on the vascular system are likely superimposed on the underlying pathophysiological alterations caused by septic shock. Interestingly, high doses of norepinephrine (> 0.3 µg/kg/min) partially reversed the vascular decoupling phenomenon in the radial arteries, though not in the femoral arteries, suggesting a regional variation in the drugs effects [26, 27]. Clinical studies also showed that decreasing norepinephrine doses in vasoplegic patients can lead to a reduction in PP, but not in SV, which could be attributed to an increase in arterial compliance [27]. Furthermore, in experimental rat models, phenylephrine infusion increased PP disproportionately compared to SV, with PP rising by 58% while SV increasing by only 21% [28]. These findings were also observed in critically ill patients, in whom introduction or increase in norepinephrine increased peripheral PP by far more than CO [20]. This finding was attributed to the fact that vasopressors alter both arterial compliance and PWA, making PP less correlated to SV than during a fluid challenge [20].

### Arterial line site

The site of arterial catheter may also influence PP recordings [29]. Radial arteries, the most common site for arterial catheters, have smaller diameters and more muscular walls compared to central arteries, which may impair pressure transmission [11]. This effect can be further amplified by vasopressor-induced vasoconstriction, resulting in lower radial SAP and MAP readings. In septic shock patients treated with norepinephrine, Kim et al. reported lower radial compared to femoral MAP (85 vs. 91 mmHg) and PP (62 vs. 65 mmHg) [30]. Of note, similar gradients have been observed in cardiac surgery patients, with lower radial MAP and PP compared to femoral measurements (radial MAP: 72 mmHg, radial PP: 38 mmHg; femoral MAP: 76 mmHg, femoral PP: 53 mmHg) [31].

Despite all the confounders (Fig. 2), peripheral PP may still be a promising bedside indicator of SV, which could be particularly important during the early stages of shock resuscitation. A low PP may indicate low SV, often due to hypovolemia or myocardial dysfunction, common features of shock states. Such patients may benefit from early echocardiographic and fluid responsiveness assessment, to guide therapy (i.e., fluid boluses) aiming at restoring tissue perfusion. Conversely, a higher PP may help rule out very low SV, especially when accompanied by low DAP, suggesting vasoplegia as the predominant cause of circulatory dysfunction. In these cases, norepinephrine administration to restore DAP could improve global hemodynamics and tissue perfusion.

## Clinical uses of pulse pressure

Although many clinicians would intuitively use PP as a surrogate for SV during bedside assessments, robust epidemiological data to validate this practice remain scarce. One study including 228 critically ill patients evaluated to which extent peripheral PP could be used as a surrogate of CO for assessing the effects of fluid challenge [24]. In these patients, CO was significantly increased in 24% of the patients, and the changes in CO were moderately correlated with changes in PP (*r* =.56, *p* <.0001). In the multivariate analysis, changes in peripheral PP were significantly related to changes in SV and age. Interestingly, a fluid-induced increase in PP of > 17% allowed detecting a fluid-induced increase in CO of > 15% with a specificity of 85%. However, the modest global area under receiving operating curve of 0.78 and the presence of false negatives significantly limits the utility of PP changes as a standalone of SV increase during a dynamic maneuver as a fluid challenge.

**Fig. 4:**
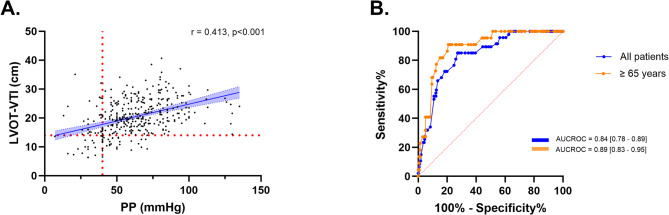
(**A**) Relationship between pulse pressure and the left ventricular outflow tract velocity time integral (LVOT-VTI). (**B**) Diagnostic accuracy of pulse pressure to predict a low LVOT-VTI (< 14 cm)

On the other hand, a secondary analysis of a Swedish echocardiographic cohort study including 359 critically ill patients [32] demonstrates a non-linear relationship between PP and SV, assessed by left ventricular outflow tract-velocity time integral (LVOT-VTI) (Fig. 4, Panel A; unpublished data). However, PP thresholds commonly used in clinical practice (e.g., < 40 mmHg) predicted low SV (LVOT-VTI < 14 cm) with high specificity (> 90%). Notably, the predictive performance of PP improved substantially in patients older than 65 years (Fig. 4, Panel B), underscoring the potential of PP as an initial tool for identifying hemodynamic patterns in resource-limited settings. As epidemiological principles suggest, when a diagnostic test is highly specific, a positive result (i.e., low PP) can effectively rule in the target condition (i.e., low SV), thereby highlighting the clinical relevance of PP.

Other clinical applications of pulse pressure have been suggested in different contexts. For instance, it has been used to predict cardiac contractility improvement during venoarterial extracorporeal membrane oxygenation (VA-ECMO) and incorporated into ECMO weaning protocols [33]. Pulse pressure variation (PPV), measured during positive pressure ventilation, is a well-established predictor of fluid responsiveness and has further utility in estimating dynamic arterial elastance. This novel metric integrates PPV and stroke volume variation to predict the arterial pressure response to volume loading or guide the safe de-escalation of vasopressor therapy [34].

Additionally, the gradient between the arterial dicrotic notch and systolic pressure has been proposed as an early marker of cardiovascular deterioration in patients with septic shock undergoing pharmacologic heart rate control with beta-blockers [35]. Finally, other uses have not yet been validated by research but make physiological sense, such as using PP as a safety limit to guide fluid removal, or to identify deleterous impact of increased pressurization during mechanical ventilation.

## Future directions

A couple of ongoing studies are expected to provide more relevant data on the potential usefulness of the relationship between PP and SV to be considered for clinical decisions: the ANDROMEDA-SHOCK-2 [36], and the large observational cross-sectional study ANDROMEDA-PEGASUS [NCT06737614]. These investigations will provide critical insights into the confounding factors, predictive boundaries, and overall reliability of PP as a decision-making tool in shock resuscitation. The former, a large randomized controlled study, is testing a resuscitation strategy targeting capillary refill time but where initial resuscitative interventions are taken based on PP. This is particularly relevant since a recent British study demonstrated that a minority of ICU patients could be subjected to a bedside echocardiography during the first hours after ICU admission [37]. By refining the application of PP in guiding therapeutic strategies, these studies may contribute to improving individualized patient care and resuscitation outcomes.

## Conclusion

Peripheral PP, a readily available bedside variable, holds promise as a surrogate marker of SV in critically ill patients. However, its interpretation requires careful consideration of arterial stiffness, vasomotor tone, and vasoactive therapies. Further research is needed to refine the utility of PP in hemodynamic profiling and guiding individualized resuscitation strategies.

## Data Availability

No datasets were generated or analysed during the current study.

## Abbreviations

CO: cardiac output
SV: stroke volume
PP: pulse pressure
MAP: mean arterial pressure
SAP: systolic arterial pressure
DAP: diastolic arterial pressure
HR: heart rate
PWA: pulse wave amplification
LVOT-VTI: left ventricle outflow tract – velocity time integral
